# Rubber-Filler Interactions in Polyisoprene Filled with In Situ Generated Silica: A Solid State NMR Study

**DOI:** 10.3390/polym10080822

**Published:** 2018-07-25

**Authors:** Silvia Borsacchi, Umayal Priyadharsini Sudhakaran, Lucia Calucci, Francesca Martini, Elisa Carignani, Massimo Messori, Marco Geppi

**Affiliations:** 1 Italian National Council for Research—Institute for the Chemistry of OrganoMetallic Compounds, CNR-ICCOM, via G. Moruzzi 1, 56124 Pisa, Italy; silvia.borsacchi@cnr.it (S.B.); lucia.calucci@pi.iccom.cnr.it (L.C.); 2 Department of Chemistry and Industrial Chemistry, University of Pisa, via G. Moruzzi 13, 56124 Pisa, Italy; umayal28@gmail.com (U.P.S.); francesca.martini@for.unipi.it (F.M.); elisa.carignani@for.unipi.it (E.C.); 3 Department of Engineering “Enzo Ferrari”, University of Modena and Reggio Emilia, via P. Vivarelli 10/1, 41125 Modena, Italy; massimo.messori@unimore.it

**Keywords:** rubber, silica, sol-gel, bound-rubber, ^1^H relaxation times, ^13^C CP-MAS, FID analysis

## Abstract

In this paper we used high- and low-resolution solid state Nuclear Magnetic Resonance (NMR) techniques to investigate a series of polyisoprene samples filled with silica generated in situ from tetraethoxysilane by sol-gel process. In particular, ^1^H spin-lattice and spin-spin relaxation times allowed us to get insights into the dynamic properties of both the polymer bulk and the bound rubber, and to obtain a comparative estimate of the amount of bound rubber in samples prepared with different compositions and sol-gel reaction times. In all samples, three fractions with different mobility could be distinguished by ^1^H *T*_2_ and ascribed to loosely bound rubber, polymer bulk, and free chain ends. The amount of bound rubber was found to be dependent on sample preparation, and it resulted maximum in the sample showing the best dispersion of silica domains in the rubber matrix. The interpretation of the loosely bound rubber in terms of “glassy” behaviour was discussed, also on the basis of ^1^H *T*_1_ and *T*_1*ρ*_ data.

## 1. Introduction

The use of inorganic fillers is a well consolidated practice for conferring and/or improving the most various technologically relevant properties, such as mechanical, optical, thermal, etc., to polymeric materials [1]. In the field of rubbers, and especially in the tyre industry, silica has been increasingly used as filler thanks to the good results obtained, especially in tread compounds, in terms of increased traction, lower rolling resistance (and thus lower vehicle fuel consumption), and good abrasion resistance [2,3,4]. In spite of the scientific progress achieved and of an extensive practical use, it is still quite difficult to precisely define the composition-process-performance relation in filler-polymer, and in particular in silica-rubber composites. It is commonly accepted that a crucial role is played by the complex balance between filler-filler and filler-polymer interfacial interactions, and, in particular, that a good dispersion of the filler in the organic matrix is a necessary condition for a well-performing composite [5]. Preparation methods are usually designed and tuned for optimizing the dispersion of the filler in the rubber. In the case of silica-rubber composites, the hydrophilic character of silica, poorly compatible with the hydrophobic rubber, and the tendency to silica-silica aggregation are obstacles to be overcome. There are three main approaches to the preparation of silica-rubber composites: (1) direct mixing of preformed silica and rubber in the melt state under strong shearing forces; (2) in situ polymerization, in solution, of the rubber in the presence of silica; (3) in situ generation of silica in the presence of rubber, often in emulsion, by sol-gel process [6]. The third method, also exploited for preparing the materials investigated in this work, has recently gained attention for the good results obtained with several polymers. Silica is obtained from the hydrolysis and condensation of alkoxysilanes, often tetraethoxysilane (TEOS), occurring under mild conditions. One of the attractive characteristics of this approach is that the amount and morphology of silica could potentially be controlled by suitably tuning the reaction conditions of the sol-gel process, such as type and amount of precursor and catalyst, reaction time, and temperature [7,8]. Moreover silane coupling agents, often used for improving the interfacial interactions between filler and rubber, can be easily introduced.

In a previous work by some of us we investigated the interrelation between preparation conditions, structure, and mechanical reinforcement in a series of vulcanized composites of isoprene rubber (IR) and silica generated in situ via sol-gel from TEOS [9]. In particular, three different initial TEOS contents were considered and for each of them the sol-gel reaction was stopped at different times, before proceeding with the vulcanization. By combining ^29^Si solid state Nuclear Magnetic Resonance (NMR), Scanning Electron Microscopy (SEM), swelling experiments, and uniaxial tensile tests, it was found that all the three factors (i.e., TEOS content, duration of the sol-gel process, and addition of the coupling agent) strongly affect structure, formation kinetics, morphology, and dispersion of silica particles in the rubber, as well as the mechanical properties of the final composites. 

^1^H low-resolution solid state NMR has been widely used to characterize polymer-filler composites [6,10,11,12,13,14,15,16,17,18,19,20,21]. In particular, spin-spin relaxation times (*T*_2_) are usually exploited to investigate polymer-filler interactions and the formation of bound rubber, while spin-lattice relaxation times in the laboratory (*T*_1_) and the rotating (*T*_1*ρ*_) frame can give insights into polymer motions (typically segmental motions above *T*_g_) occurring in the MHz and kHz regimes, respectively. On the other hand, ^13^C high-resolution solid state NMR can be very precious in characterizing the structure of the polymer, for what concerns monomeric sequences, conformational properties as well as chain packing [22].

In this work, we focused on the characterization of the polymeric fraction in this wide set of composite samples by exploiting ^13^C and ^1^H solid state NMR techniques. We mainly aimed at comparing the dynamic behaviour of the polymer and the amount of formed bound rubber (i.e., the fraction of polymer present at the interface with the silica experiencing stiffening at a molecular level) in differently prepared composites. The preparation conditions of the sample were found to not substantially affect the dynamics of the polymeric bulk in the MHz and kHz frequency regimes, involving chain segmental reorientations, and, in part, cooperative motions. On the other hand, the amount of bound rubber was found to depend on the preparation conditions, resulting maximum in a sample which was found by SEM to exhibit the best dispersion of silica particles. The bound rubber identified in all the composite samples resulted to be “loosely bound” to the silica surface. This was discussed in terms of its possible behaviour as a “glassy layer”: our results support the hypothesis that the bound rubber is characterized by a remarkable increase of the anisotropy of the segmental chain motions, but not by a reduction of their characteristic frequencies.

## 2. Materials and Methods

### 2.1. Samples Preparation

All reagents and solvents were purchased from Sigma-Aldrich (Milan, Italy) and used without further purification. The Isoprene Rubber (IR) polymer had a 97% content of cis units, a viscosity-average molecular mass of 2.3 × 10^6^ g/mol, a glass transition temperature of 206 K, and a density of 0.91 g/cm^3^. Silica/isoprene rubber composites were prepared following the procedure described in Reference [9]. Briefly, IR polymer was dissolved in toluene at the refluxing temperature. Tetraethoxysilane, H_2_O, and ethanol (1:4:4 molar ratios), dibutyltin laurate (catalyst for the sol-gel process, 2 wt % relative to TEOS), and dicumyl peroxide (vulcanizing agent, 1 wt % relative to TEOS) were added after cooling at room temperature. The sol-gel conversion of TEOS to silica was let to proceed by heating the mixtures at 80 °C, under magnetic stirring, for different times. After chosen reaction times, all volatile reagents/products were evaporated and samples were vulcanized at 150 °C for 20 min under 150 bar pressure. The obtained and analysed samples are coded as IRV_*x*_*y*, where IRV stands for vulcanized IR, *x* is the nominal silica content (in phr) expected in the case of full TEOS hydrolysis and condensation and *y* the sol-gel reaction time in minutes. The actual silica content and the degree of conversion of TEOS to silica, as well as SEM micrographs of the investigated materials, are reported in Reference [9]. The swollen sample (IRV_30_180sw) was prepared by immersing IRV_30_180 in toluene-d_8_, which was daily replaced with a fresh one, for 96 h.

### 2.2. Solid State NMR

^13^C spectra and ^1^H spin-lattice relaxation times in the laboratory (*T*_1_) and in the rotating (*T*_1*ρ*_) frame were recorded using a two-channel Varian Infinity Plus 400 spectrometer (Palo Alto, CA, USA), operating at 400.03 and 100.55 MHz for hydrogen-1 and carbon-13 nuclei, respectively, equipped with a 7.5 mm CP-MAS (Cross Polarization-Magic Angle Spinning) probehead. ^13^C spectra were recorded under MAS (3–4 kHz frequency) and High Power Decoupling (HPD) from ^1^H nuclei, exploiting both Direct Excitation (DE) and CP pulse sequences. The DE-MAS spectra were recorded with a recycle delay of 2 s. For CP-MAS spectra, a contact time of 10 ms and a recycle delay of 3.5 s were used. In both the experiments, 22,000 transients were accumulated. TMS and hexamethylbenzene were used as primary and secondary chemical shift references, respectively. All the experiments were carried out at room temperature using air as spinning gas. ^1^H *T*_1_ and *T*_1*ρ*_ were measured in static conditions at room temperature. *T*_1_ was obtained by applying the saturation recovery pulse sequence, with variable recovery delay ranging from 0.001 to 5 s. *T*_1*ρ*_ was measured by exploiting the variable spin-lock time pulse sequence, with a spin-lock time varying between 0.1 and 50 ms, a spin-lock field of 35 kHz, and a recycle delay of 3.5 s.

^1^H spin-spin relaxation times *T*_2_^′^s were obtained by combining solid echo (SE) and Hahn echo (HE) experiments carried out in low-resolution conditions, at room temperature, on a Varian XL-100 (Palo Alto, CA, USA) interfaced with a Stelar DS-NMR (Mede, Italy) acquisition system, equipped with a 5 mm probehead, working at a ^1^H Larmor frequency of 25 MHz. The ^1^H 90° pulse duration was 4 μs and a recycle delay of 0.1 s was used. In the case of SE, the delay between the 90° pulses was 14 μs and 1000 transients were recorded. For HE, variable echo delays (τ) ranging from 50 μs to 25 ms were used and 500 transients accumulated. For each sample a “reconstructed“ Free Induction Decay (FID) was obtained by matching the points of the FID recorded with SE from 100 to 160 μs with those of the decay curve (magnetization intensity vs. 2τ) obtained from HE in the same time interval. The reconstructed FIDs were analysed with an in-house package developed within the Mathematica [23] environment.

## 3. Results

### 3.1. ^13^C Solid State NMR Spectra

**Figure 1:**
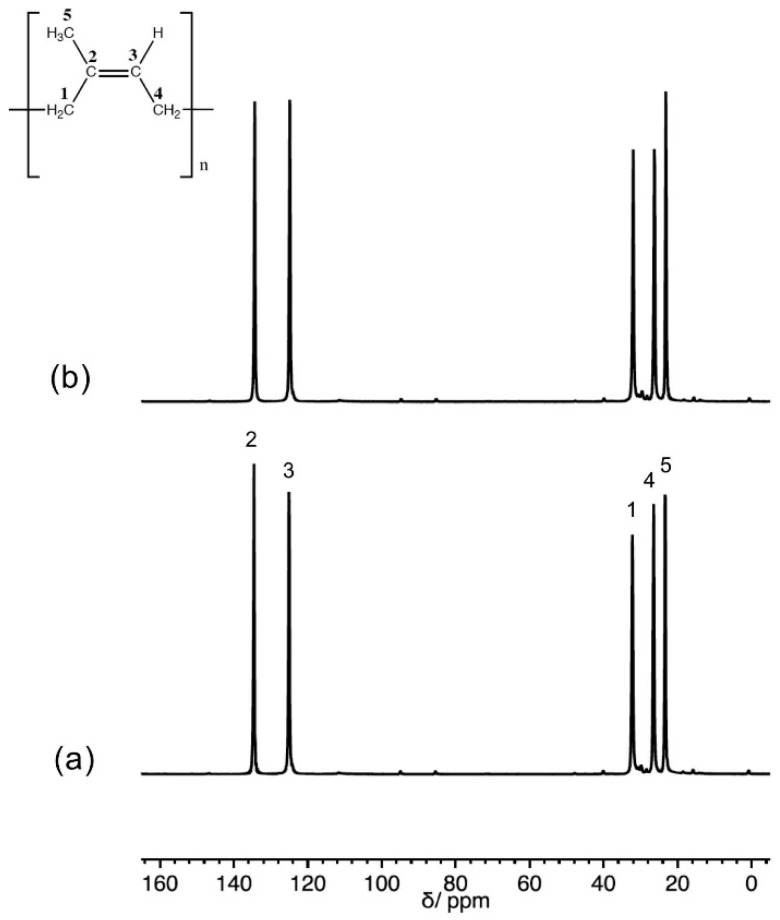
^13^C DE-MAS spectra of IRV (**a**) and IRV_30_180 (**b**).

Figure 1 shows the ^13^C Solid State NMR spectra of pristine IRV polymer and of one polymer/filler composite (IRV_30_180). The ^13^C spectra reported in Figure 1 were recorded using the DE-MAS pulse sequence and a short recycle delay between consecutive transients, conditions that favour the signals of ^13^C nuclei with short spin-lattice relaxation times *T*_1_^′^s, usually associated with a high degree of mobility. In all samples at room temperature (about 90 degrees above *T*_g_) the polymer was in a very mobile rubber phase. Indeed all expected carbon signals typical of 1,4-*cis*-polyisoprene were observed, with a very good signal to noise ratio. Very weak signals ascribable to monomeric units in trans configuration could be observed at about 15, 28, 40, and 124 ppm. On the other hand, neither in DE nor in CP-MAS spectra (not shown) could signals ascribable to ethoxy groups of TEOS be observed, indicating a complete hydrolisation of these groups.

### 3.2. ^1^H Spin-Lattice Relaxation Times T_1_ and T_1ρ_

In order to compare the effects of the presence and the amount of silica on the dynamic behaviour of the polymer, we measured ^1^H *T*_1_ and *T*_1*ρ*_ on the composites obtained with the longest sol-gel reaction time (Table 1). On the basis of the silica/polymer weight ratio and of the silica condensation degree previously determined [9], from 95% to 98% of the ^1^H nuclei belong to IRV, and therefore the *T*_1_ and *T*_1*ρ*_ values can be safely considered to arise from such nuclei. The spin-lattice relaxation in the laboratory frame resulted to be mono-exponential, thus a single *T*_1_ value was measured, indicating that the polymeric fraction was homogeneous on an approximately 10 nm spatial scale in all samples [24]. Moreover, all samples showed a substantially identical *T*_1_, suggesting that the fast (MHz regime) interconformational polymer chain motions were not sensitive to the presence of the silica filler. On the other hand, a bi-exponential spin-lattice relaxation in the rotating frame was observed for all samples. A multi-exponential *T*_1*ρ*_ decay has been already reported [25] for monophasic amorphous polymers above *T*_g_, and in order to obtain dynamic information, it is useful to calculate the inverse of the population weighted rate average (PWRA), defined as:

(1) $$\mathrm{PWRA}=\frac{1}{100}\underset{i}{\sum }\frac{w_{i}}{T_{1\rho ,i}}$$

where the sum runs over the number of exponentials, and the i-th exponential has a percentual weight *w*_i_, and a relaxation time *T*_1*ρ*,i_ [26]. The 1/PWRA measured for the IRV_xx_180 samples were very similar, and also similar to that of IRV, indicating that the dynamic processes of the polymer with characteristic frequency in the regime of kHz were, like those in the MHz regime, substantially unaffected by the presence of the filler.

### 3.3. ^1^H Spin-Spin Relaxation Times T_2_

**Figure 2:**
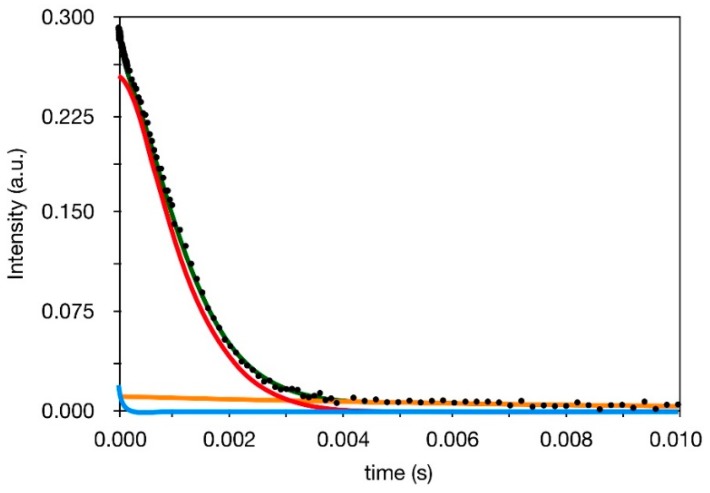
Example of fit of a reconstructed ^1^H FID: experimental data (black points), total fitting function (green line), exponential function with *T*_2_ = 70 μs (blue line), Weibullian function (red line), and exponential function with *T*_2_ = 10 ms (orange line).

In order to get insights into the presence of polymeric fractions with different mobility, and in particular to try to detect and characterize the bound rubber, we measured ^1^H spin-spin relaxation times *T*_2_ on the whole set of samples available. In Figure 2, as an example, the proton FID of IRV_50_60, reconstructed as described in the Experimental section from the SE and HE experiments, is shown together with its fitting. In all cases the reconstructed FID could be well fitted with the following linear combination of two exponential and one Weibullian functions:

(2) $$M\left(t\right)=M_{a}\left(0\right)\left(\exp\left(-t/T_{2a}\right)\right)+M_{b}\left(0\right)\left(\exp{\left(-(t/T_{2b}\right)}^{\alpha }\right)+M_{c}\left(0\right)\left(\exp\left(-t/T_{2c}\right)\right)$$

*T*_2i_ is the *T*_2_ of the i-th relaxation component, *M*_i_ (0) is the amplitude of the same component, which, suitably normalized, represents the percentage of ^1^H nuclei associated with *T*_2i_. α is the shape parameter of the Weibullian component (ranging from 1 to 2, limit values corresponding to exponential and Gaussian functions, respectively).

**Figure 3:**
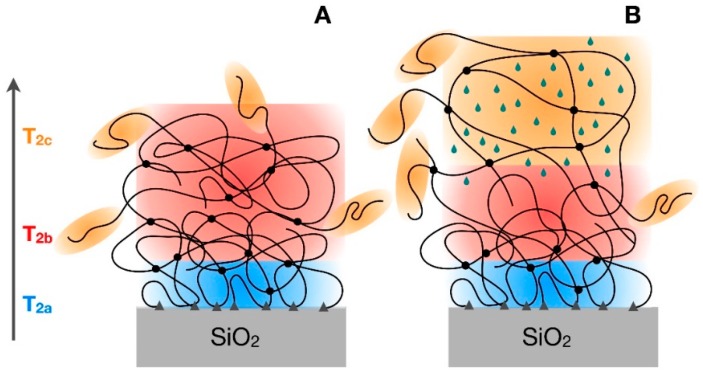
Sketch of the different regions of the polymer in the unswollen (**A**) and swollen (**B**) states, as detected by ^1^H FID analysis. Blue, red, and orange colours refer to the “a”, “b”, and “c” components of the FID.

A first series of fits carried out on the reconstructed FIDs without any constraint showed almost equal values of *T*_2a_, *T*_2c_, and α for all samples. Therefore, in order to reduce the correlation among fitting parameters, and to facilitate a physically meaningful interpretation of the results, we fixed *T*_2a_, *T*_2c_, and α at their best-fitting values of 70 μs, 10 ms, and 1.5, respectively. The remaining best-fitting parameters so obtained are reported in Table 2, where, in particular, it can be observed that *T*_2b_ always assumes very similar values between 1.3 and 1.7 ms in all samples, apart from the swollen one. The three different *T*_2_ values identify three polymeric fractions characterized by clearly different dynamic properties [27]. The “b” fraction, with a relatively long *T*_2_ of about 1.5 ms, is the most abundant and it can be surely identified with the bulk of the rubber; the use of a Weibullian function to describe this component was necessary to take phenomenologically into account the distribution of mobility situations present in the bulk, as often reported in the literature [28,29,30]. On the other hand, the small fraction of protons characterized by the longest *T*_2_ of 10 ms (“c” component) can be safely ascribed to the free chain ends of the polymer, experiencing a larger mobility. The most interesting proton fraction is that with the shortest *T*_2_ of 70 μs (“a” component). These protons clearly belong to polymer chains experiencing a much more restricted mobility with respect to the polymer bulk, which in principle can be identified with both physical entanglements occurring in the polymer bulk and bound rubber that is the polymer fraction closely interacting with the inorganic filler. Pristine IRV shows a very small amount (2%) of protons in restricted mobility, fully ascribable to physical entanglements, suggesting that most of the protons contributing to the “a” component in the other samples belong to the bound rubber. This is further confirmed by the analysis of IRV_30_180 swollen in deuterated solvent (Table 2). In the presence of solvent, the weight of the “a” component remains similar to that of IRV_30_180, in agreement with the scarce permeability of the bound rubber, while the polymer bulk is largely swollen and the chain mobility strongly increased, as shown by the increase of *T*_2b_ and the weight transfer from “b” to “c” component. This situation is sketched in Figure 3.

**Figure 4:**
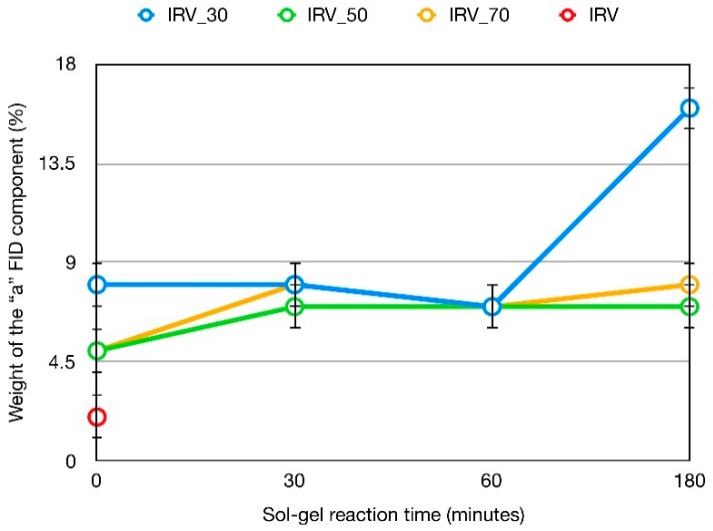
*w*_a_ (%) vs. sol-gel reaction time for the samples IRV_*x* prepared at different nominal silica content *x*, as obtained by analysis of reconstructed ^1^H FID’s.

The trends of *w*_a_ (%) with sol-gel reaction times for the series of samples with different nominal content of silica are shown in Figure 4. By looking at the values at time zero, the slightly larger *w*_a_ found in IRV_30_0 with respect to IRV_50_0 and IRV_70_0 corresponds to a previously found higher TEOS conversion degree (36% vs. 19 and 20%), and suggests a better silica dispersion in IRV_30 already at the early reaction times. From 0 to 30 min *w*_a_ of IRV_50 and IRV_70 increases and then remains almost constant, in qualitative agreement with the trends of silica contents. On the contrary, in the case of IRV_30 a large increase of *w*_a_ is observed from 60 to 180 min of sol-gel reaction time, which corresponds to its maximum increase of silica content. The peculiarly high value of *w*_a_ in IRV_30_180, indicating that this sample has the highest amount of bound rubber, suggests that this combination of initial TEOS content and sol-gel reaction time particularly favours the formation of bound rubber. A further confirmation of this can be obtained by looking at the intensity of the ^29^Si CP-MAS spectra reported in our previous paper [9]. Considering that all the samples have very similar silica condensation degrees, and assuming that the ^29^Si magnetization is mainly built by transfer of the magnetization of silanol protons, the total signal intensity of the spectrum should be in principle roughly proportional to the actual silica content. However, Simonutti et al., [15] have proposed that the ^29^Si signal intensity of CP-MAS spectra of IR-silica composites is also affected by magnetization transfer from protons of the bound rubber. Indeed, in the spectrum of IRV_30_180, the ratio between the total integral of the spectrum and the actual silica content largely exceeds that of the other samples, in agreement with both the contribution to the ^29^Si CP spectrum from bound rubber protons and with the biggest amount of bound rubber present in this sample. Noticeably, this result agrees with SEM images [9], which highlighted that IRV_30_180 has the best dispersion of small silica aggregates in the rubber matrix. 

Although the weights of the different components reported above have been used only for obtaining comparative results, it is worthy to stress that the experimental method here employed (basically SE acquired with an echo delay of 14 μs) can bring to biased results, and in particular, to an underestimate of the weights of the most rigid fractions. To overcome this problem, when quantitative results are strictly necessary, two main approaches can be applied, consisting either in the extrapolation to zero of the weights obtained by SE as a function of the echo delay or in the use of the magic sandwich echo (MSE) technique. Both these methods were applied to a representative sample and they agreed in finding an underestimation of about 20% for the *w*_a_ values here determined.

## 4. Discussion

The value of 70 μs found for the *T*_2_ of protons belonging to the bound rubber deserves a deeper discussion. In the literature, values ranging from 10–20 μs (characteristic of very rigid solid domains) to hundreds of μs were reported for ^1^H *T*_2_ associated to the bound rubber. These results have been often interpreted in terms of “bond strength” of the rubber bound to the filler. Values of *T*_2_ of the order of 10–20 μs have been associated to “tightly bound rubber” (i.e., chains experiencing a very restricted overall mobility), as distinguished from “loosely bound rubber”, experiencing a larger degree of mobility and associated to ^1^H *T*_2_ values of the order of several tens/hundreds of μs [11,31]. In the frame of this approach, our experimental data indicate the presence of loosely bound rubber only. On the other hand, the mobility of the bound rubber, as detected by ^1^H *T*_2_, has been also discussed in comparison with the mobility of the pure polymer. Roughly speaking, the bound rubber can be seen as a “glassy layer” (i.e., a temperature shift of the relaxation curves can be hypothesized so to bring the *T*_2_ measured for the bound rubber to coincide with the *T*_2_ measured for the pure polymer at a lower temperature) [20,32,33,34,35]. In the case of loosely bound rubber, the effective temperature of the bound rubber would coincide with the NMR *T*_g_ of the pure polymer, where *T*_2_ steeply increases with increasing temperature and segmental motions with characteristic frequencies of the order of tens of kHz take place. However, different papers, often based on data arising from different experimental techniques, described the behaviour of this “glassy layer” in a somehow divergent way [10]. We try here to give a small contribution to this discussion, by simultaneously interpreting *T*_2_ and *T*_1_/*T*_1*ρ*_ data. If bound rubber really behaved as the polymer at its NMR *T*_g_, this would also imply the temperature shift of *T*_1_ and *T*_1*ρ*_ curves; then the presence of segmental motions in the kHz regime should favour spin-lattice relaxation in the rotating frame (decreasing ^1^H *T*_1*ρ*_ values) and disfavour spin-lattice relaxation in the laboratory frame (increasing ^1^H *T*_1_ values). In our case, this is not experimentally observed even in IRV_30_180, where the maximum amount of bound rubber is present. Therefore, we support the hypothesis, in agreement with Golitsyn et al. [10], that the segmental motions in the loosely bound rubber preserve similar characteristic frequencies as those of the polymeric bulk much above *T*_g_ (not affecting *T*_1_ and *T*_1*ρ*_). At the same time, because of the interactions with the filler surface, these motions preserve a strictly local character, showing a larger anisotropy, and therefore bringing to a significant increase of the residual homonuclear dipolar coupling and to a corresponding decrease of *T*_2_.

## Figures and Tables

**Table 1:** ^1^H *T*_1_ and *T*_1*ρ*_ values of the indicated samples, measured at a Larmor frequency of 400.03 MHz. In the case of *T*_1*ρ*_, both the population weighted rate average (PWRA) (Equation (1)) and the values of the two components of the detected bi-exponential relaxation with their weight percentages are reported. Errors of about ±10 and 1 ms were estimated for *T*_1_ and 1/PWRA, respectively.

Sample	*T*_1_ (s)	*T*_1*ρ*,1_ (ms)	*w*_1_	*T*_1*ρ*,2_ (ms)	*w*_2_	1/PWRA (ms)
IRV	0.67	4.5	15	13.2	85	10
IRV_30_180	0.65	3.4	14	13.2	86	9
IRV_50_180	0.66	3.3	15	13.0	85	9
IRV_70_180	0.65	4.5	19	14.2	81	10

**Table 2:** Results of the fitting of the transverse magnetization decay curves, reconstructed from SE and HE experiments as described in the text, by the function reported in equation 2: *T*_2a_ and *T*_2c_ were kept fixed at the values of 70 μs and 10 ms, respectively. *w*_i_ (%) = 100*M_i_(0)/*M*(0) with i = a, b, or c. The values of the actual silica content for the different samples are also reported. Errors of about ±1% and 0.05 ms could be estimated for *w*_i_ (%) and *T*_2b_, respectively.

Sample	Silica Content (phr) ^b^	*w*_a_ (%)	*T*_2b_ (ms)	*w*_b_ (%)	*w*_c_ (%)
IRV	0	2	1.33	91	7
IRV_30_0	10.9	8	1.72	84	8
IRV_30_30	16.1	8	1.72	86	6
IRV_30_60	17.9	7	1.52	87	6
IRV_30_180	23.9	16	1.56	79	5
IRV_50_0	9.6	5	1.41	88	7
IRV_50_30	34.5	7	1.35	90	3
IRV_50_60	39.0	7	1.34	89	4
IRV_50_180	43.0	7	1.36	87	6
IRV_70_0	14.3	5	1.35	86	9
IRV_70_30	48.2	8	1.44	90	2
IRV_70_60	49.7	7	1.35	87	6
IRV_70_180	49.5	8	1.38	88	4
IRV_30_180sw ^a^	23.9	21	2.2	46	33

^a^ This sample was swollen in toluene-d_5_ as described in the Experimental section. ^b^ Actual silica content as determined from thermogravimetric measurements (data taken from Reference [9]).
